# Supplementary material for: Statistical characteristics of analytical studies published in Peruvian medical journals from 2021 to 2022: A methodological study
Source: PLoS One. 2024 Jul 3;19(7):e0306334. doi: 10.1371/journal.pone.0306334 (PMC11221636; doi:10.1371/journal.pone.0306334)
Supplement: Attachment — Submitted filename: Reviews.pdf [file pone.0306334.s002.pdf]

Comments:

The study provides a valuable contribution to understanding the state of statistical reporting in Peruvian biomedical articles. Despite its limitations, the findings highlight critical areas for improvement and set the stage for future research. By addressing the identified deficiencies and implementing the recommended improvements, the quality and credibility of Peruvian biomedical research can be significantly enhanced.

Although this new study is interesting I have some reservations as below:

- In the abstract section, the keywords are not arranged in alphabetical order which needs to be rewritten.
- On page number 12 section, regarding the Data collection process and variables, the authors did not mention the (NNA, DGL, DFG, and ATR).
- Page number 13, the section on statistical analysis is not attractive to the readers. The authors give very limited information for the analysis.
- The explanations provided for the methodologies used in adjusted analyses were often insufficient. This includes unclear descriptions of the statistical techniques used to account for confounding factors and biases, making it difficult for readers to assess the robustness and validity of the results.
- Page number 13, " We reported medians and 25th and 75th) percentiles" The authors did not mention where these percentiles are given in any table or figure
- The information given in the result section is very limited, this section needs to add more description.
- The length of sentences in this research article exceeded more than 20 words, which is not attractive to readers internationally.
