# Supplementary material for: Statistical characteristics of analytical studies published in Peruvian medical journals from 2021 to 2022: A methodological study
Source: PLoS One. 2024 Jul 3;19(7):e0306334. doi: 10.1371/journal.pone.0306334 (PMC11221636; doi:10.1371/journal.pone.0306334)

PLOS ONE

Statistical characteristics of analytical studies published in Peruvian medical journals from 2021 to 2022: a methodological study

--Manuscript Draft--

**Manuscript Number:**	PONE-D-24-15400
**Article Type:**	Research Article
**Full Title:**	Statistical characteristics of analytical studies published in Peruvian medical journals from 2021 to 2022: a methodological study
**Short Title:**	Statistical characteristics of analytical studies published in Peruvian medical journals from 2021 to 2022
**Corresponding Author:**	Alvaro Taype-Rondan<br>Universidad San Ignacio de Loyola<br>Lima, PERU
**Keywords:**	Statistical Data Analysis; Sample Size; Research Design; Biomedical Research; Peru (source: MeSH)
**Abstract:**	Objective<br><br>While statistical analysis plays a crucial role in medical science, some published studies might have utilized suboptimal analysis methods, potentially undermining the credibility of their findings. Critically appraising analytical approaches can help elevate the standard of evidence and ensure clinicians and other stakeholders have trustworthy results on which to base decisions. The aim of the present study was to examine the statistical characteristics of original articles published in Peruvian medical journals in 2021 - 2022.<br><br>Design and setting<br><br>We performed a methodological study of articles published between 2021 and 2022 from nine medical journals indexed in SciELO-Peru, Scopus, and Medline. We included original articles that conducted analytical analyses (i.e., association between variables). The statistical variables assessed were: statistical software used for analysis, sample size, and statistical methods employed (measures of effect), controlling for confounders, and the method employed for confounder control or epidemiological approaches.<br><br>Results<br><br>We included 313 articles (ranging from 11 to 77 across journals), of which 67.7% were cross-sectional studies. While 90.7% of articles specified the statistical software used, 78.3% omitted details on sample size calculation. Descriptive and bivariate statistics were commonly employed, whereas measures of association were less common. Only 13.4% of articles (ranging from 0% to 39% across journals) presented measures of effect controlling for confounding and explained the criteria for selecting such confounders.<br><br>Conclusion<br><br>This study revealed important statistical deficiencies within analytical studies published in Peruvian journals, including inadequate reporting of sample sizes, absence of measures of association and confounding control, and suboptimal explanations regarding the methodologies employed for adjusted analyses.
**Order of Authors:**	Natalia Nombera-Aznaran
	David Guevara-Lazo
	Daniel Fernandez-Guzman
	Alvaro Taype-Rondan

Powered by Editorial Manager® and ProduXion Manager® from Aries Systems Corporation

Opposed Reviewers:	
Additional Information:	
Question	Response
**Financial Disclosure**<br><br>Enter a financial disclosure statement that describes the sources of funding for the work included in this submission. Review the submission guidelines for detailed requirements. View published research articles from *PLOS ONE* for specific examples.<br><br>This statement is required for submission and **will appear in the published article** if the submission is accepted. Please make sure it is accurate.<br><br>**Funded studies**<br>Enter a statement with the following details:<br>• Initials of the authors who received each award<br>• Grant numbers awarded to each author<br>• The full name of each funder<br>• URL of each funder website<br>• Did the sponsors or funders play any role in the study design, data collection and analysis, decision to publish, or preparation of the manuscript?<br><br>Did you receive funding for this work?	The author(s) received no specific funding for this work.
**Competing Interests**<br><br>Use the instructions below to enter a competing interest statement for this submission. On behalf of all authors, disclose any competing interests that could be perceived to bias this work—acknowledging all financial support and any other relevant financial or non-financial competing interests.<br><br>This statement is required for submission and **will appear in the published article** if the submission is accepted. Please make sure it is accurate and that any funding	The authors have declared that no competing interests exist.

sources listed in your Funding Information later in the submission form are also declared in your Financial Disclosure statement.<br><br>View published research articles from *PLOS ONE* for specific examples.<br><br>**NO authors have competing interests**<br><br>Enter: *The authors have declared that no competing interests exist.*<br><br>**Authors with competing interests**<br><br>Enter competing interest details beginning with this statement:<br><br>*I have read the journal's policy and the authors of this manuscript have the following competing interests: [insert competing interests here]*<br><br>* typeset	
**Ethics Statement**<br><br>Enter an ethics statement for this submission. This statement is required if the study involved:<br><br>• Human participants<br>• Human specimens or tissue<br>• Vertebrate animals or cephalopods<br>• Vertebrate embryos or tissues<br>• Field research<br><br>Write "N/A" if the submission does not require an ethics statement.<br><br>General guidance is provided below. Consult the submission guidelines for detailed instructions. **Make sure that all information entered here is included in the Methods section of the manuscript.**	N/A

**Format for specific study types**<br><br>**Human Subject Research (involving human participants and/or tissue)**<br>• Give the name of the institutional review board or ethics committee that approved the study<br>• Include the approval number and/or a statement indicating approval of this research<br>• Indicate the form of consent obtained (written/oral) or the reason that consent was not obtained (e.g. the data were analyzed anonymously)<br><br>**Animal Research (involving vertebrate animals, embryos or tissues)**<br>• Provide the name of the Institutional Animal Care and Use Committee (IACUC) or other relevant ethics board that reviewed the study protocol, and indicate whether they approved this research or granted a formal waiver of ethical approval<br>• Include an approval number if one was obtained<br>• If the study involved *non-human primates*, add *additional details* about animal welfare and steps taken to ameliorate suffering<br>• If anesthesia, euthanasia, or any kind of animal sacrifice is part of the study, include briefly which substances and/or methods were applied<br><br>**Field Research**<br><br>Include the following details if this study involves the collection of plant, animal, or other materials from a natural setting:<br>• Field permit number<br>• Name of the institution or relevant body that granted permission	
**Data Availability**<br><br>Authors are required to make all data underlying the findings described fully available, without restriction, and from the time of publication. PLOS allows rare exceptions to address legal and ethical concerns. See the PLOS Data Policy and FAQ for detailed information.	Yes - all data are fully available without restriction

Powered by Editorial Manager® and ProduXion Manager® from Aries Systems Corporation

A Data Availability Statement describing where the data can be found is required at submission. Your answers to this question constitute the Data Availability Statement and **will be published in the article**, if accepted.<br><br>**Important:** Stating 'data available on request from the author' is not sufficient. If your data are only available upon request, select 'No' for the first question and explain your exceptional situation in the text box.<br><br>Do the authors confirm that all data underlying the findings described in their manuscript are fully available without restriction?	
**Describe where the data may be found in full sentences. If you are copying our sample text, replace any instances of XXX with the appropriate details.**<br><br>• If the data are **held or will be held in a public repository**, include URLs, accession numbers or DOIs. If this information will only be available after acceptance, indicate this by ticking the box below. For example: *All XXX files are available from the XXX database (accession number(s) XXX, XXX.*).<br>• If the data are all contained **within the manuscript and/or Supporting Information files**, enter the following: *All relevant data are within the manuscript and its Supporting Information files.*<br>• If neither of these applies but you are able to provide **details of access elsewhere**, with or without limitations, please do so. For example:<br><br>*Data cannot be shared publicly because of [XXX]. Data are available from the XXX Institutional Data Access / Ethics Committee (contact via XXX) for researchers who meet the criteria for access to confidential data.*<br><br>*The data underlying the results presented in the study are available from (include the name of the third party*	All data are fully available without restriction.

Powered by Editorial Manager® and ProduXion Manager® from Aries Systems Corporation

and contact information or URL).<br>• This text is appropriate if the data are owned by a third party and authors do not have permission to share the data.<br><br>* typeset	
Additional data availability information:	

Powered by Editorial Manager® and ProduXion Manager® from Aries Systems Corporation

Emily Chenette, PhD
PLOS ONE

Dear Emily Chenette, PhD,

I am writing to submit my research paper titled "Statistical characteristics of analytical studies published in Peruvian medical journals from 2021 to 2022," for consideration for publication in PLOS ONE. I believe that this work aligns with the scope and standards of your journal, and it addresses a critical aspect of statistical analysis in the field of medicine.

The primary motivation behind this study is the recognition that the credibility of medical research heavily relies on the appropriateness and accuracy of statistical methods employed. With this in mind, our research aimed to critically appraise the statistical characteristics of original articles published in Peruvian medical journals. By examining the analytical approaches utilized in these studies, we sought to enhance the evidence standard, ultimately providing clinicians and stakeholders with more reliable results for informed decision-making.

In our scoping review of 313 articles from nine medical journals indexed in SciELO-Peru, Scopus, and Medline, we uncovered significant deficiencies in the statistical reporting of these studies. Notably, a substantial proportion of articles omitted crucial details such as sample size calculations (78.3%), measures of association (87.6%), and adequate control for confounders in adjusted analysis methodologies (86.6%). Furthermore, our findings underscored the prevalence of suboptimal explanations regarding adjusted analysis methodologies in the examined articles.

The importance of our research lies in its potential to contribute to the improvement of statistical practices in medical research, particularly in the Peruvian context. By shedding light on these deficiencies, we hope to stimulate discussions within the scientific community and encourage researchers, reviewers, and journal editors to prioritize robust statistical methods for more credible and impactful findings.

Given the alignment of our study with the mission of PLOS ONE and the significance of the topic within the medical research community, I believe that our work is suitable for publication in your journal. Thank you for considering our submission. I look forward to the opportunity for our research to be reviewed by the esteemed experts associated with PLOS ONE.

Sincerely,

Alvaro Taype-Rondan
MD
EviSalud - Evidencias en Salud, Lima, Perú
alvaro.taype.r@gmail.com
+51 970001517

Manuscript Click here to access/download;Manuscript;Manuscript (1).docx

Statistical characteristics of analytical studies published in Peruvian medical journals from 2021 to 2022: a methodological study

Authors

Natalia Nombera-Aznaran[1], David Guevara-Lazo[1], Daniel Fernandez-Guzman[2], Alvaro Taype-Rondán[3,4]

Affiliation

[1] Facultad de Medicina Humana, Universidad Peruana Cayetano Heredia, Lima, Perú.

[2] Carrera de Medicina Humana, Universidad Científica del Sur, Lima, Perú.

[3] Universidad San Ignacio de Loyola, Unidad de Investigación para la Generación y Síntesis de Evidencias en Salud, Lima, Perú.

[4] EviSalud - Evidencias en Salud, Lima, Perú.

*Correspondence

- Name: Alvaro Taype-Rondan
- Mail: alvaro.taype.r@gmail.com
- Phone: +51 970001517

Author contributions

ATR conceived the idea. NNA and DGL collected the data. DFG performed the statistical analysis. All authors participated in the manuscript's writing and revision. All authors approved the final version of the manuscript.

Founding

This study was self-funded by the authors.

Conflicts of interest

The authors do not report having any conflicts of interest.

Abstract

Objective: While statistical analysis plays a crucial role in medical science, some published studies might have utilized suboptimal analysis methods, potentially undermining the credibility of their findings. Critically appraising analytical approaches can help elevate the standard of evidence and ensure clinicians and other stakeholders have trustworthy results on which to base decisions. The aim of the present study was to examine the statistical characteristics of original articles published in Peruvian medical journals in 2021 - 2022.

Design and setting: We performed a methodological study of articles published between 2021 and 2022 from nine medical journals indexed in SciELO-Peru, Scopus, and Medline. We included original articles that conducted analytical analyses (i.e., association between variables). The statistical variables assessed were: statistical software used for analysis, sample size, and statistical methods employed (measures of effect), controlling for confounders, and the method employed for confounder control or epidemiological approaches.

Results: We included 313 articles (ranging from 11 to 77 across journals), of which 67.7% were cross-sectional studies. While 90.7% of articles specified the statistical software used, 78.3% omitted details on sample size calculation. Descriptive and bivariate statistics were commonly employed, whereas measures of association were less common. Only 13.4% of articles (ranging from 0% to 39% across journals) presented measures of effect controlling for confounding and explained the criteria for selecting such confounders.

Conclusion: This study revealed important statistical deficiencies within analytical studies published in Peruvian journals, including inadequate reporting of sample sizes, absence of measures of association and confounding control, and suboptimal explanations regarding the methodologies employed for adjusted analyses.

Keywords: Statistical Data Analysis; Sample Size; Research Design; Biomedical Research; Peru (source: MeSH)

Introduction

Health research plays a crucial role in understanding the afflictions that impact humanity and in making informed healthcare decisions.[1] However, when research fails to adhere to best practices in methodology and statistics, the credibility of its results may be compromised. While the primary responsibility lies with the study authors, the efforts of reviewers and journal editors play a vital role in refining scientific articles before their publication.[2]

Given the importance of ensuring adequate statistical methodologies, previous studies have examined this subject within medical journals. A study performed in the Journal of the American Medical Association showed that in 2010 around half of papers used advanced adjusted regression models.[3] Conversely, a study in a Colombian medical journal highlighted substandard reporting of statistical regression models.[4] Moreover, an examination of statistical methodologies employed in orthopedic papers revealed that in one-third of the papers an alternative analysis should have been conducted and in 17% choosing for a different statistical analysis had the potential to change the conclusions.[5]

While statistical methodology holds universal importance in medical research, it poses specific challenges in emerging research contexts. Countries that are in the process of developing their healthcare research capacities require local researchers to best understand existing gaps and priorities for improvement. Peru has witnessed a surge in research activity in recent decades,[6,7] yet there are opportunities to enhance methodological rigor. Analyzing statistical practices in Peruvian medical journals can offer insights tailored to the country's needs, considering the ongoing development of both the educational system and publication culture. Identifying areas of strength in statistical analysis, as well as aspects that require

strengthening, will help Peruvian researchers, reviewers, and editors, as well as those in similar contexts where local research is on the rise, to elevate the standard of evidence. This ensures that clinicians and other stakeholders have reliable results upon which to base their decisions. Hence, the objective of this study was to analyze the statistical characteristics of original analytical articles published in Peruvian medical journals.

Methods

Design and Data Source

We conducted a methodological study encompassing all original analytical articles published in Peruvian medical journals indexed in SciELO-Peru, Scopus, or Medline, between 2021 and 2022.

We included all nine medical journals that were indexed in SciELO-Peru, Scopus, or Medline during data extraction (April 2023). These were: Acta Médica Peruana, Revista Médica Herediana, Revista de la Facultad de Medicina Humana, Horizonte médico, Revista del Cuerpo Médico del Hospital Nacional Almanzor Aguinaga Asenjo, Revista de Neuro-Psiquiatría, Revista Peruana de Ginecología y Obstetricia, Revista de Gastroenterología del Perú, and Revista Peruana de Medicina Experimental y Salud Pública.

Subsequently, we examined the official websites of these journals to scrutinize every article published in their volumes corresponding to the years 2021 and 2022. Our inclusion criteria encompassed articles of original studies performed on human subjects, which have incorporated analytical assessments (i.e., associations between variables using any bivariate association analyses). We excluded secondary studies such as systematic reviews or scoping reviews, as well as validation studies.

Data collection process and variables

For article selection and data extraction, each article was reviewed independently by two of the authors (NNA and DGL). In cases of doubts or discrepancies, these were resolved together with a third author (DFG or ATR). Data was extracted into an Excel spreadsheet.

The general variables extracted from each article included: journal name, year of publication, field of study (clinical [articles that focus on the diagnosis, treatment, and management of individual patients or groups of patients] or public health [these articles address topics such as disease prevention, health promotion, health policy, epidemiology, health disparities, environmental health, and social determinants of health]), study design, time between submission and acceptance of the manuscript, number of authors, and presence of any Peruvian authors.

The statistical variables assessed were: statistical software used for analysis, sample size, and statistical methods employed: measures of effect (linear regression beta coefficient [β], odds ratio [OR], prevalence ratio [PR], relative risk [RR], hazard ratio [HR]), controlling for confounders, and the method employed for confounder control (statistical [based on p-values or other statistical methods] or epidemiological approaches [involving theoretical rationale and clinical relevance from literature]). These variables were chosen according to previous studies.[4,5,8–10] This data was gathered by thoroughly reviewing each article, with particular focus on the methods and results sections, although additional sections were consulted when required.

Statistical Analysis

We imported the collected data into R software, version 4.1.0 (R Foundation for Statistical Computing, Vienna, Austria), where we conducted all analyses and generated graphics. Since numerical variables exhibited a non-normal distribution, we reported medians and the 25th and 75th percentiles. Categorical variables were presented as absolute and relative frequencies.

Results

In the volumes corresponding to 2020 and 2021 of the nine medical journals indexed in SciELO-Peru, a total of 1361 published articles (original and non-original articles) were initially identified. Subsequently, 564 original articles were evaluated. After applying inclusion and exclusion criteria, 313 studies (150 in 2021 and 163 in 2022), which have incorporated analytical assessments, were included. Of these, 69.6% focused on clinical research, 67.7% were cross-sectional studies, 83.1% had at least one author affiliated with an institution in Peru, and the median time between submission and acceptance of articles was 92.5 days (Table 1).

We found different patterns regarding the statistical analysis in the methodology section. The majority of the studies (90.1%) specified the statistical software used in their analyses. 78.3% of the studies did not mention their sample size or statistical power calculation. Among the studies that calculated sample size (n = 57), 77% performed calculations based on the study design, but 56.1% did not report the use of parameters based on previous studies. Also, 61.4% did not report the statistical power used in the calculations.

Regarding analysis methods, we included studies that performed any bivariate association analyses (hypothesis tests), but only 42.5% included association analyses with regressions or generalized linear models, while other analyses (complex survey sampling, diagnostic tests, time series analysis, geospatial analysis, and machine learning analysis) were even less common. Furthermore, 47.6% did not present measures of effect, 23.03% only presented crude measures of effect, 15.3% presented adjusted measures of effect but did not explain the criteria used for selecting confounders; and 13.74% presented adjusted measures of effect and explained the criteria used for selecting confounders (Table 2).

Regarding the statistical analysis software used, there was a preference for SPSS (42.5%) and Stata (38.0%). Concerning the normality assessment of numerical variables, most studies (78.6%) did not include or did not report how it was performed, while Kolmogorov-Smirnov (9.9%) and Shapiro-Wilk (8.9%) tests were the most frequently mentioned. Regarding bivariate tests, the most commonly used ones were the Chi-Square test (66.1%), Student's t-test (31.3%), Fisher's test (25.2%), and Mann-Whitney U test (22.4%). Regarding regression models, Poisson regression (19.5%) and logistic regression (15.7%) were the most frequent models. Finally, concerning the visual presentation of results, most studies (58.8%) did not use graphics to illustrate their findings (**Figure 1**).

Across the included journals, the percentage of articles lacking effect measures varied from 28.3% to 86.7%, the percentage of articles that displayed adjusted measures varied from 0% to 46.3%, and the percentage of articles that presented adjusted measures with an explanation of confounder selection criteria varied from 0% to 39.0% (**Figure 2**).

Discussion

Sample size and statistical power

Whether the sample is collected for the study or obtained from a pre-existing database, determining the sample size is crucial to ensure the minimum number of subjects necessary to detect specific associations. Similarly, in the case of secondary data analysis, it is important to assess whether non-significant findings are due to a lack of statistical power.

However, a significant proportion of original articles (78%) show deficiencies in specifying the calculation of sample size or statistical power. Even when the sample size is addressed, essential information used for its computation is often omitted, hindering the replicability of the analysis. Similar findings were reported by Kumar et al., in their examination of the trend and status of the application of statistics in 300 biomedical research articles. They found that, despite the mention of sample size in 95% of the articles, its reproducibility was limited to only 7%. In the remaining 93% of articles, neither the precise mention of the sample size estimation technique nor an adequate description was provided.[11] Likewise, a review of 448 studies published in the Balkan Medical Journal revealed a reporting of sample size in 8.1% of cross-sectional studies, 13.8% in cohorts, and 16% in clinical trials.[12] Even in high-impact general medical journals, only one-third of articles adequately describe sample size calculations.[13]

Measures of effect

Our study found that 47% of articles lacked measures of effect. A parallel trend was identified in a study that assessed 60 observational studies in high-impact occupational medicine and health journals.[14] Findings indicated that, although all studies reported outcome events or summary measures, only 60% provided unadjusted estimates, and merely 43.3% included confounder-adjusted estimates.[14] Similarly, an examination of the articles in the Journal of

the American Medical Association between 1990 and 2010 uncovered a significant increase in incorporating epidemiologic statistics, reaching almost 30%.[3] This highlights a gap in reporting standards, emphasizing the need for improved consistency in presenting both unadjusted and adjusted estimates in observational research. In contrast, a comprehensive analysis of The Biomedica journal in Colombia, covering approximately 113 studies from 2000 to 2017, revealed an impressive inclusion of nearly 90% reporting measures of association coefficients.[4]

Measuring the strength of observed associations between a given risk factor and an outcome is an important goal in epidemiological and clinical research.[15] Effect sizes—both unstandardized and standardized from individual studies—should be reported for inclusion in systematic reviews and meta-analyses.[16] However, our analysis revealed that only 13.4% of the articles provided criteria for selecting confounding variables, which are integral to adjusted analyses. These can be chosen based on established causal relationships, statistical associations (p-value < 0.05), or an epidemiological approach. In our study, confounding variables were primarily selected through the last two methods.

Adequate management of confounding variables is essential for isolating the true impact of the exposure under investigation (causal inference).[17–20] Specifying criteria for selecting confounding variables in an adjusted model analysis ensures systematic identification and control, enhancing the reliability of research findings. Despite explicit reporting recommendations for handling missing data within the STrengthening the Reporting of OBservational studies in Epidemiology (STROBE) guidelines,[21] we found no articles adhering to these recommendations. Failing to handle missing data appropriately may lead

to incorrect conclusions and potential harm to patients if clinical decisions are based on incomplete or biased information.[16]

Even among high-impact medical journals, deficiencies persist in reporting STROBE items. For instance, high-impact occupational medicine and health journals reported all STROBE items in only 63% of articles, while otorhinolaryngology journals adequately reported a mean of 51.4% of STROBE items.[14,22] Our findings underscore the need for enhanced reproducibility and quality reporting in observational studies published in Peruvian journals. Therefore, addressing this issue should be a central point for both authors and editors when reporting or reviewing observational studies.

Inadequate presentation of the data

Our analysis identified a frequent oversight in the presentation of data within the examined articles. The mention of the statistical software was absent in 9.9% of the articles. Furthermore, 48.6% of the articles presented tables/figures that included p-values but in which the statistical tests used were not specified.

Tables play a crucial role in presenting research results. Even when presented independently, they should include essential elements necessary for communicating information, such as population parameters, alongside confidence intervals, p-values, and the utilized test statistics.[23] Inclusion of these components in tables facilitates interpretation of results for readers. The presentation of p-values and confidence intervals enables clinicians to discern the statistical significance and understand the differences in outcomes between two groups.[24] While p-values indicate statistical significance, confidence intervals establish the threshold for the magnitude of the effect. Both of these statistical measures are integral in the

interpretation of clinical outcomes. Therefore, ensuring their inclusion in tables enhances the clarity and meaningfulness of research findings.

Peruvian journals

Peru encounters significant challenges regarding the representation of its journals in key international databases like Scopus and Medline. At the time of the study, only two Peruvian journals, "Revista Peruana de Medicina Experimental y Salud Pública" and "Revista de Gastroenterología del Perú," are indexed in both Medline and Scopus. This aligns with the current national situation, where we have approximately 92 health science journals, with around 95% remaining unindexed.[25]

This scenario is significantly impacted by crucial factors, stemming from a shortage of researchers and a prevalent inclination to publish their research i[illegible]urnals indexed in international databases such as Scopus or Medline.[26] Additional challenges include insufficient financial support and promotion for national medical journals. Collectively, these factors diminish visibility and recognition both regionally and internationally.

Our study highlights significant concerns regarding the methodological reporting of statistical processes in articles published in Peruvian journals. Introducing a statistical review of the submitted articles as an integral part of the editorial process, as suggested in our study, could be a replicable strategy for enhancing the reporting quality of research articles in diverse international contexts and improving confidence in their results.[27]

National journals are important to spread studies of local interest, which may be of special interest for local practitioners and stakeholders.[28] Nevertheless, as revealed by our study,

studies published in local Peruvian medical journals need to improve their statistical report, which ultimately may underpin their credibility and usage.

Efforts to identify and improve the statistical deficiencies of Peruvian medical journals can foster a more inclusive and comprehensive representation of diverse perspectives in the global scientific community. By recognizing the challenges faced by Peruvian journals and proposing solutions, our study aims to contribute to a broader conversation on strengthening the integrity and impact of research from developing regions, ultimately fostering a more interconnected and equitable landscape for scientific knowledge dissemination.

Limitations and strengths

The findings of this study shed light on critical aspects of statistical reporting in Peruvian biomedical articles. However, some limitations should be taken into account when interpreting the results. Firstly, the temporal scope of the study, which focused on publications from 2021 and 2022 to reflect the current state of the topic, but may not be generalizable to different time periods, as statistical reporting may change over an extended time. Additionally, while the study reviewed statistical reporting, it did not thoroughly cover all aspects of research methodology.

It is essential to recognize that despite the limitations reported in the medical Peruvian journals, they do not fully represent the medical research produced in Peru, since the majority of Peruvian biomedical articles are published in international journals, which were not the focus of this investigation.[6,29]

Turning to the strengths of the study, to our knowledge, this is the first study conducted in Peru and within Spanish journals assessing statistical reporting in medical literature.

Furthermore, a thorough article analysis was carried out in duplicate to ensure optimal data extraction.

Conclusion

Our study reveals significant weakness in analytical studies published in SciELO-indexed Peruvian journals including inadequate reporting of sample size, absence of measures of association and adjusted analyses, suboptimal documentation of the methods used for adjusted analyses, and inadequate presentation of the data. These findings underscore the importance of reinforcing reporting statistical standards in biomedical research. Moreover, the study highlights the importance for collaborative efforts between the researchers and the editorial team. By enhancing these aspects, Peruvian journals can improve the quality of research production.

References

1. Schünemann HJ, Reinap M, Piggott T, et al. The ecosystem of health decision making: from fragmentation to synergy. *Lancet Public Health*. 2022;7(4):e378-e390. doi:10.1016/S2468-2667(22)00057-3
2. Glonti K, Cauchi D, Cobo E, Boutron I, Moher D, Hren D. A scoping review on the roles and tasks of peer reviewers in the manuscript review process in biomedical journals. *BMC Med*. 2019;17:118. doi:10.1186/s12916-019-1347-0
3. Arnold LD, Braganza M, Salih R, Colditz GA. Statistical Trends in the Journal of the American Medical Association and Implications for Training across the Continuum of Medical Education. *PLOS ONE*. 2013;8(10):e77301. doi:10.1371/journal.pone.0077301
4. Fernández-Niño JA, Hernández-Montes RI, Rodríguez-Villamizar LA. Reporting of statistical regression analyses in Biomédica: A critical assessment review. *Biomed Rev Inst Nac Salud*. 2018;38(2):173-179. doi:10.7705/biomedica.v38i0.3648
5. Parsons NR, Price CL, Hiskens R, Achten J, Costa ML. An evaluation of the quality of statistical design and analysis of published medical research: results from a systematic survey of general orthopaedic journals. *BMC Med Res Methodol*. 2012;12(1):1-9. doi:10.1186/1471-2288-12-60
6. Sevillano-Jimenez J, Carrión-Chambilla M, Espinoza-Lecca E, Mori-Quispe E, Contreras-Pulache H, Moya-Salazar J. A bibliometric analysis of 47-years of research on public health in Peru. *Electron J Gen Med*. 2023;20(4):em488. doi:10.29333/ejgm/13103
7. Fernandez-Guzman D, Montesinos-Segura R, Caira-Chuquineyra B, et al. Producción científica en Scopus de los institutos de salud especializados públicos de Perú, 2010-2022. *Rev Cuerpo Méd Hosp Nac Almanzor Aguinaga Asenjo*. 2023;16(1):58-64. doi:10.35434/rcmhnaaa.2023.161.1561
8. Jaykaran, Saxena D, Solanki P, Kantharia N. Study design and statistical methods in Indian medical journals. *J Pharm Negat Results*. 2011;2(1):35. doi:10.4103/0976-9234.82983
9. Lee PH, Tse ACY. The quality of the reported sample size calculations in randomized controlled trials indexed in PubMed. *Eur J Intern Med*. 2017;40:16-21. doi:10.1016/j.ejim.2016.10.008
10. Emerson JD, Colditz GA. Use of Statistical Analysis in The New England Journal of Medicine. *N Engl J Med*. 1983;309(12):709-713. doi:10.1056/NEJM198309223091206
11. Kumar A, Kishun J, Singh U, Gaur D, Mishra P, Pandey CM. Use of appropriate statistical tools in biomedical research: Current trend & status. *Indian J Med Res*. 2023;157(4):353-357. doi:10.4103/ijmr.IJMR_809_20
12. Süt N, Ajredani M, Koçak Z. Importance of Sample Size Calculation and Power Analysis in Scientific Studies: An Example from the Balkan Medical Journal. *Balk Med J*. 2022;39(6):384-385. doi:10.4274/balkanmedj.galenos.2022.31102022
13. Charles P, Giraudeau B, Dechartres A, Baron G, Ravaud P. Reporting of sample size calculation in randomised controlled trials: review. *The BMJ*. 2009;338:b1732. doi:10.1136/bmj.b1732
14. Aghazadeh-Attari J, Mobaraki K, Ahmadzadeh J, Mansorian B, Mohebbi I. Quality of observational studies in prestigious journals of occupational medicine and health based on Strengthening the Reporting of Observational Studies in Epidemiology (STROBE) Statement: a cross-sectional study. *BMC Res Notes*. 2018;11(1):266. doi:10.1186/s13104-018-3367-9
15. Tripepi G, Jager KJ, Dekker FW, Zoccali C. Measures of Effect in Epidemiological Research. *Nephron Clin Pract*. 2010;115(2):c91-c93. doi:10.1159/000312870
16. Davis SL, Johnson AH, Lynch T, et al. Inclusion of Effect Size Measures and Clinical Relevance in

Research Papers. *Nurs Res*. 2021;70(3):222-230. doi:10.1097/NNR.0000000000000494

17. Skelly AC, Dettori JR, Brodt ED. Assessing bias: the importance of considering confounding. *Evid-Based Spine-Care J*. 2012;3(1):9-12. doi:10.1055/s-0031-1298595
18. Lipsky AM, Greenland S. Causal Directed Acyclic Graphs. *JAMA*. 2022;327(11):1083-1084. doi:10.1001/jama.2022.1816
19. Suttorp MM, Siegerink B, Jager KJ, Zoccali C, Dekker FW. Graphical presentation of confounding in directed acyclic graphs. *Nephrol Dial Transplant*. 2015;30(9):1418-1423. doi:10.1093/ndt/gfu325
20. Tennant PWG, Murray EJ, Arnold KF, et al. Use of directed acyclic graphs (DAGs) to identify confounders in applied health research: review and recommendations. *Int J Epidemiol*. 2021;50(2):620-632. doi:10.1093/ije/dyaa213
21. von Elm E, Altman DG, Egger M, Pocock SJ, Gøtzsche PC, Vandenbroucke JP. The Strengthening the Reporting of Observational Studies in Epidemiology (STROBE) Statement: Guidelines for Reporting Observational Studies. *PLoS Med*. 2007;4(10):e296. doi:10.1371/journal.pmed.0040296
22. Hendriksma M, Joosten MHMA, Peters JPM, Grolman W, Stegeman I. Evaluation of the Quality of Reporting of Observational Studies in Otorhinolaryngology - Based on the STROBE Statement. *PLOS ONE*. 2017;12(1):e0169316. doi:10.1371/journal.pone.0169316
23. Kwak SG, Kang H, Kim JH, et al. The principles of presenting statistical results: Table. *Korean J Anesthesiol*. 2021;74(2):115-119. doi:10.4097/kja.20582
24. Phillips MR, Wykoff CC, Thabane L, Bhandari M, Chaudhary V. The clinician's guide to p values, confidence intervals, and magnitude of effects. *Eye*. 2022;36(2):341-342. doi:10.1038/s41433-021-01863-w
25. BVS Perú | Biblioteca Virtual en Salud – Perú. Accessed December 15, 2023. http://www.bvs.org.pe/
26. Artigas W, Vega-Mori L. Las revistas científicas relegadas: el arduo camino de las revistas peruanas. *E-Cienc Inf*. Published online 2023. doi:10.15517/eci.v13i1.52557
27. Cobo E, Selva-O'Callagham A, Ribera JM, Cardellach F, Dominguez R, Vilardell M. Statistical Reviewers Improve Reporting in Biomedical Articles: A Randomized Trial. *PLOS ONE*. 2007;2(3):e332. doi:10.1371/journal.pone.0000332
28. Trostle J, Bronfman M, Langer A. How do researchers influence decision-makers? Case studies of Mexican policies. *Health Policy Plan*. 1999;14(2):103-114.
29. Vásquez-Uriarte K, Roque-Henriquez JC, Angulo-Bazán Y, et al. Análisis bibliométrico de la producción científica peruana sobre la COVID-19. *Rev Peru Med Exp Salud Publica*. 2021;38(2):224-231. doi:10.17843/rpmesp.2021.382.7470

Tables and figures

Table 1. General characteristics of analytical original studies published in Peruvian indexed in SciELO-Peru, 2021-2022 (n = 313)

Characteristics	Total<br>n (%)
**Journal**	
Revista de la Facultad de Medicina Humana	77 (24.6)
Revista del Cuerpo Médico del Hospital Nacional Almanzor Aguinaga Asenjo	60 (19.2)
Horizonte Médico	46 (14.7)
Revista Peruana de Medicina Experimental y Salud Pública	41 (13.1)
Acta Médica Peruana	26 (8.31)
Revista de Gastroenterología del Perú	20 (6.4)
Revista Peruana de Ginecología y Obstetricia	17 (5.4)
Revista Médica Herediana	15 (4.8)
Revista de Neuro-Psiquiatría	11 (3.5)
**Year of publication**	
2021	150 (47.9)
2022	163 (52.1)
**Study area**	
Clinical	218 (69.6)
Public health	95 (30.4)
**Study design**	
Cross-sectional	212 (67.7)
Prospective cohort	46 (14.7)
Case control	25 (8.0)
Quasi-experimental	13 (4.2)
Retrospective cohort	10 (3.2)
Randomized clinical trial	4 (1.3)
Ecological	3 (1.0)
**Time between submission and acceptance, in days***	92.5 (50.5 - 146.3)
**Number of authors***	4 (3 - 6)
**Had at least one author with Peruvian affiliation**	260 (83.1)

* Median (percentile 25 – percentile 75)

Table 2. Features of the reported methodology on statistical analyses conducted in the included studies (n = 313)

**Feature**	**Total N (%)**
**Specifies the statistical software used for the analyses**	284 (90.7)
**Mentions how the sample size calculation or statistical power was performed**	
None of them	245 (78.3)
Sample size	57 (18.2)
Statistical power	11 (3.51)
**Analysis performed***	
Present bivariate association analyses	313 (100.0)
Present descriptive analyses	311 (99.4)
Present association analyses with regressions or generalized linear models	133 (42.5)
Present analyses for surveys with complex sampling (e.g., ENDES)	11 (3.5)
Present diagnostic test analyses	11 (3.5)
Present analyses with Machine Learning	2 (0.6)
Present time series analyses	1 (0.3)
Present geospatial analyses	1 (0.3)
Perform imputation of missing data	0 (0.0)
**In tables/figures that present p-values, is it explicitly stated which test was used to calculate p-values?**	
Do not provide p-value in tables	28 (9.0)
No, provide p-value, but do not specify the test	152 (48.6)
Yes, but not for all tests	36 (11.5)
Yes, all tables/figures specify the test	97 (31.0)
**Present measures of effect?**	
Do not present measures of effect	149 (47.6)
Only present crude measures of effect	73 (23.3)
Present adjusted measures of effect, but do not explain the criteria used to select confounders**	49 (15.7)
Present adjusted measures of effect and explain the criteria used to select confounders**	42 (13.4)
**Measures of effect used (n = 164 studies that present measures of effect)**	
OR	66 (40.2)
PR	58 (35.4)
RR	19 (11.6)
HR	13 (7.9)
Beta coefficient	6 (3.7)
PR and RR	1 (0.6)
HR and OR	1 (0.6)
**Use a statistical approach for selecting variables in the adjusted model (n = 91 studies that explained this criteria)**	
Do not specify a statistical or epidemiological approach for selecting variables	67 (73.6)
P-value < 0.05 in the bivariate model	15 (16.5)
P-value < 0.20 in the bivariate model	6 (6.6)
Information criteria (Akaike or Bayesian)	2 (2.2)
Other criteria	1 (1.1)
**Use an epidemiological approach for selecting variables in the adjusted model (n = 91 studies that explained this criteria)**	19 (20.9)

*Since categories were not mutually exclusive, the total percentage can exceed 100%.

**All studies that controlled for confounders did it using adjusted regressions.

Abbreviations: ENDES: National Demographic and Family Health Survey; OR: Odds Ratio; PR: Prevalence Ratio; RR: Relative Risk; HR: Hazard Ratio.

Figure 1. Panel on software, statistics, and graphics used in the included articles.

A) Software (categories were not mutually exclusive); B) Normality assessment methods; C) Descriptive statistics; D) Bivariate statistics (hypothesis tests); E) Regression models; F) Graphics.

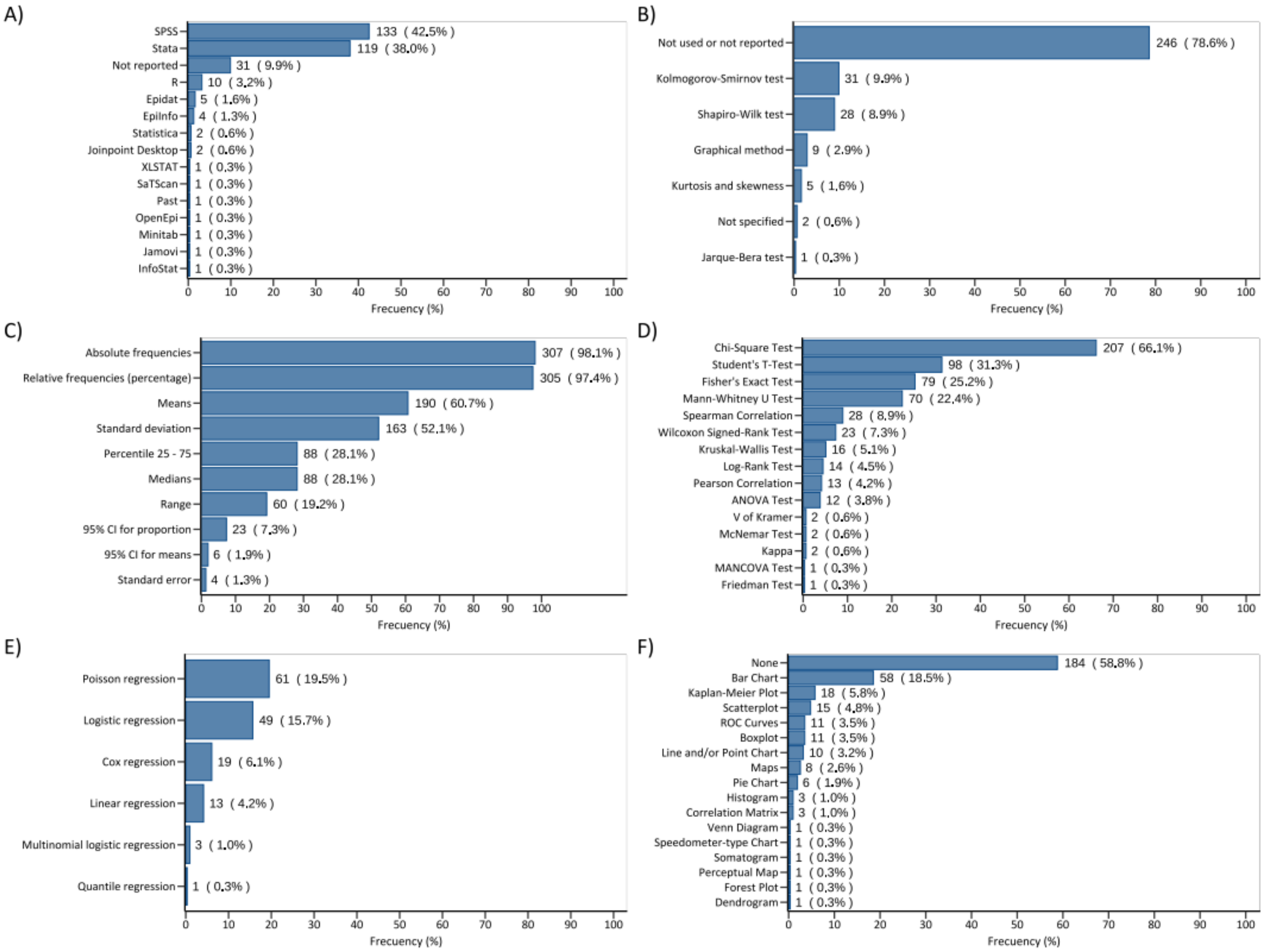

Figure 2. Calculation of effect measures in the included articles, per journal (N = 313).

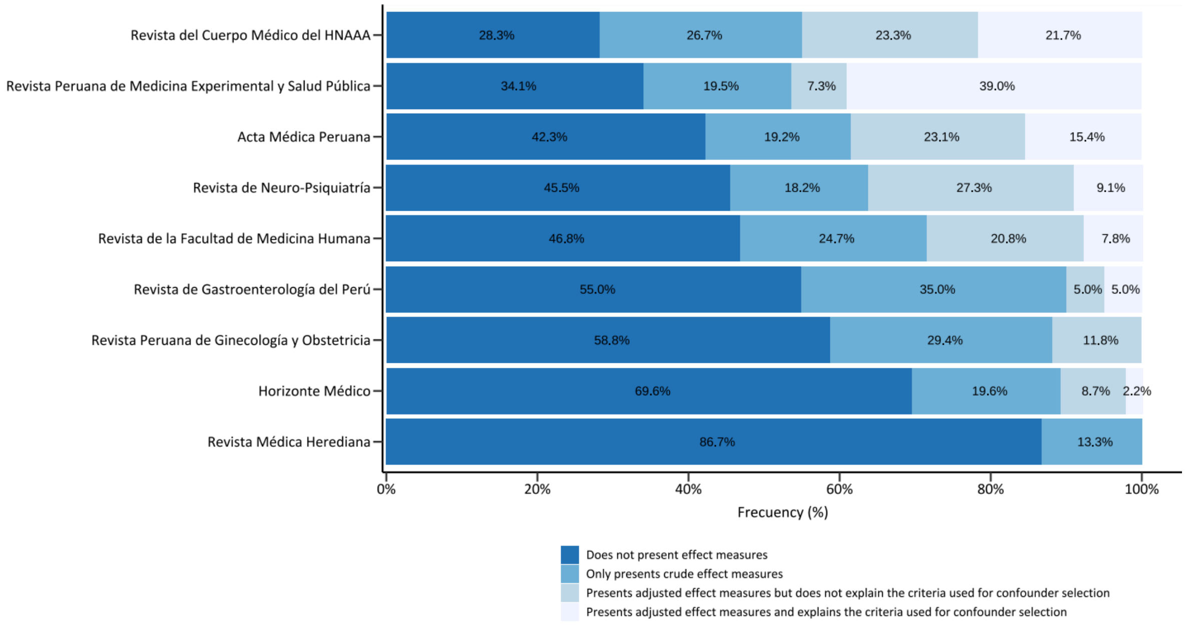

Figure 2

Click here to access/download;Figure;Figure 2.pdf

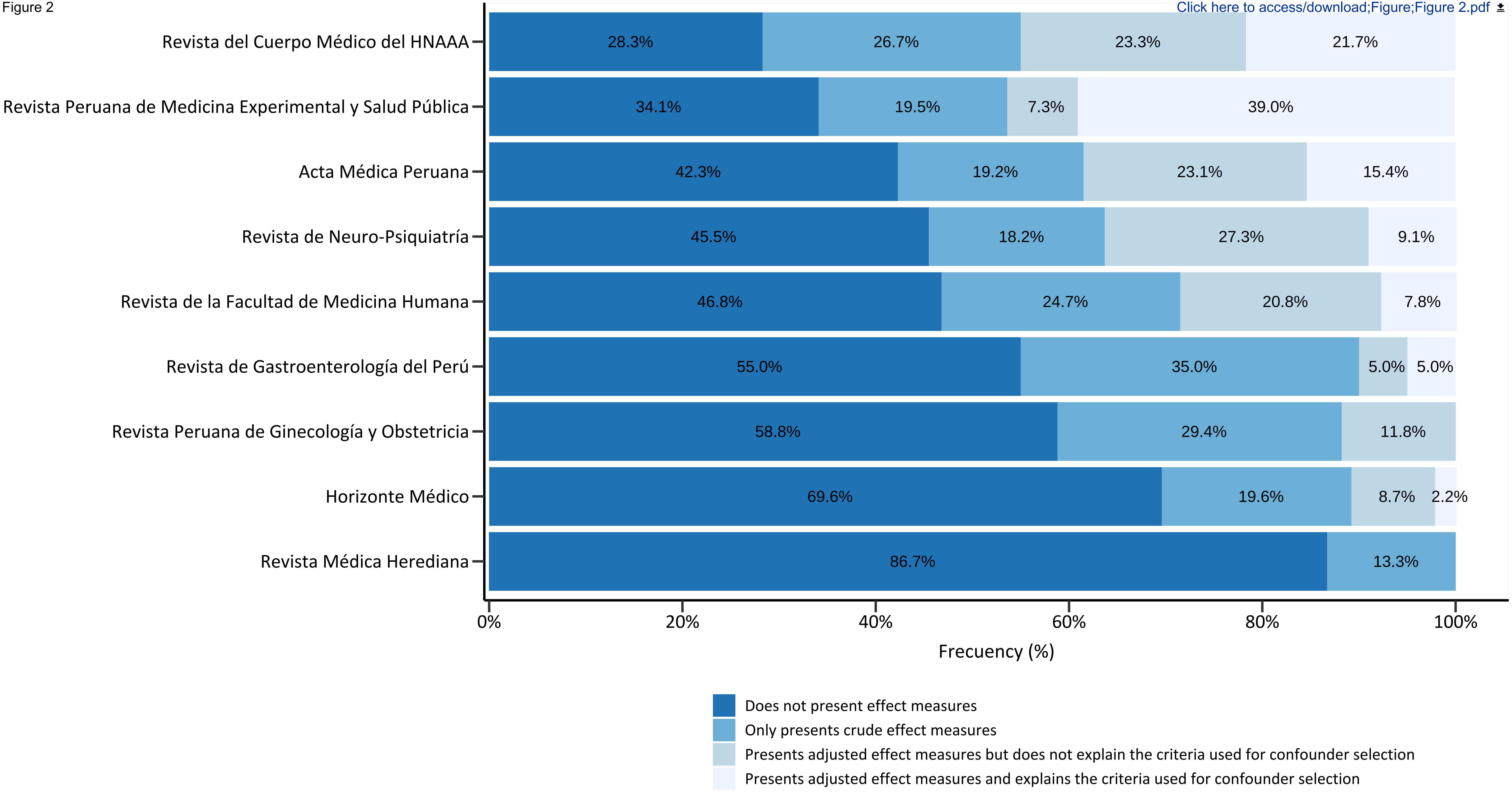

Figure 1
Click here to access/download;Figure;Figure 1.pdf

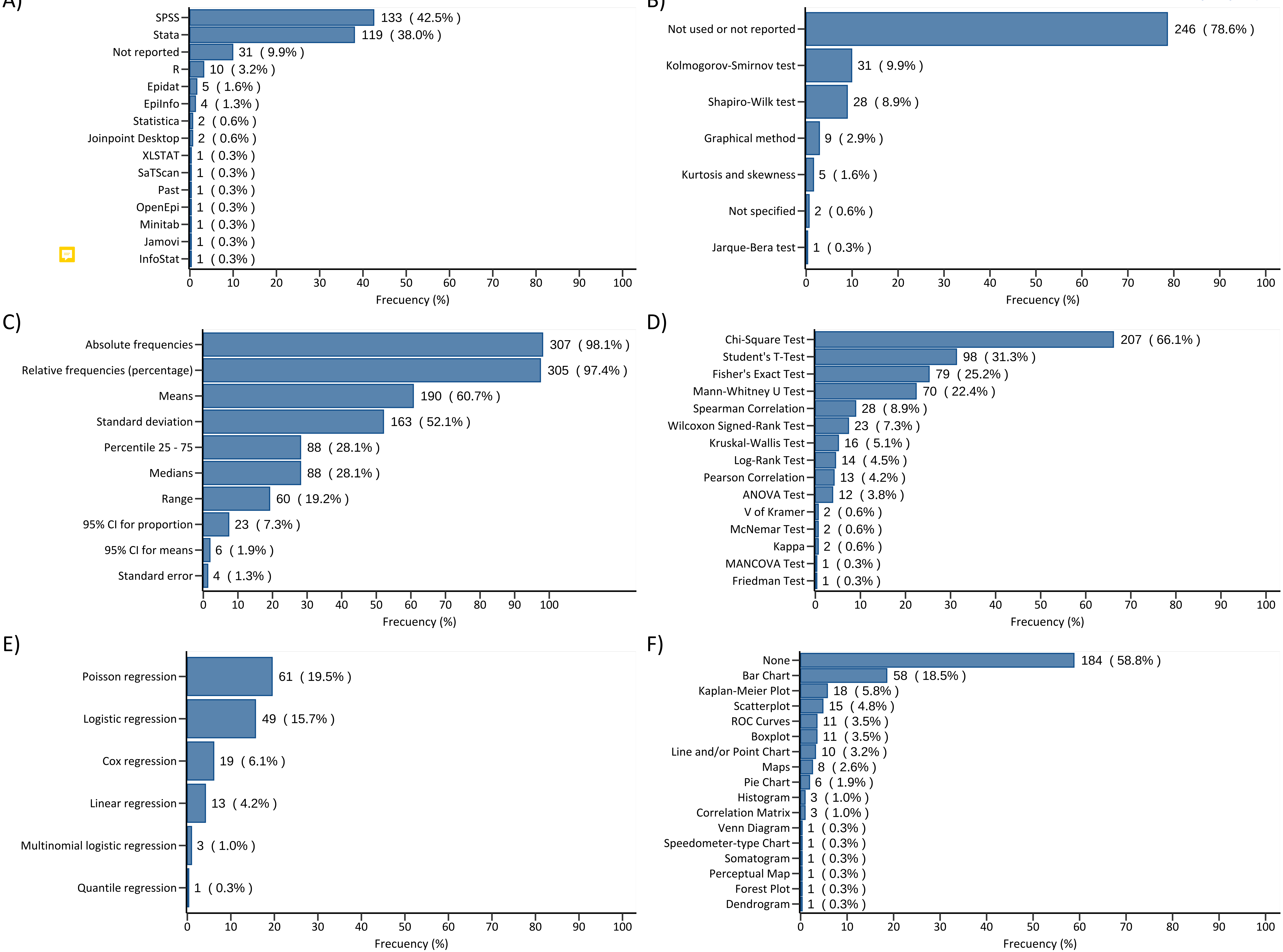

Supplement: Attachment — Submitted filename: PONE-D-24-15400-AF.pdf [file pone.0306334.s003.pdf]
